# University Diplomas in Nursing

**Published:** 1921-02-05

**Authors:** 


### THE MATRONS' AND SISTERS' DEPARTMENT.

### UNIVERSITY DIPLOMAS IN NURSING.

We were able to announce last week that the authorities of the Leeds General Infirmary had approached the Leeds University with a request for the institution of a university course for nurses crowned by a diploma. Whether the request is granted in the near future, or whether the institution of a course of study leading up to the bestowal of such diplomas has to be deferred, it is impossible to ignore the significance of the request. It testifies to the desire of voluntary hospitals managers to promote in every possible way the higher education of nurses, and is a clear proof that there is a strong demand for the more highly-educated nurses in hospitals. Nothing is more evident in the development of modern nursing than the need for those super nurses whom Florence Nightingale considered indispensable to a perfected system. While on the one hand, the call for a minimum standard of nursing education, and a minimum length of training to which all must conform has become unanimous, on the other hand, the call for women whose faculties have been developed and their minds trained beyond what is required of the average nurse echoes more loudly every day. Uniformity of system, uniformity of salary, are not compatible with the bestowal of university diplomas preceded by special courses of study.

The average nurse, capable of passing a simple examination in the theory and practice of nursing after very careful coaching extending over three years, need not possess a very high standard of education. And this is a very good thing, for so deplorably low is the standard of education in this country at present, that there would not be nearly enough trained nurses to supply vacancies were any but a very simple pass examination necessary to qualify. Should the General Nursing Council be so ill-advised as unduly to stiffen up the examination which will form the necessary passport to registration, it is tolerably certain that the majority of nurses would either decline the ordeal or fail in the attempt. The problem on which nursing education for the future can be said to hinge is whether the women of higher education, who are aspiring to take a diploma in nursing after a special course of study, should be compelled to spend three years in preparation for a simple qualifying examination which may yet involve a good deal of memorising? In our judgment it is as unreasonable to insist on every kind of nurse going through the same system of lectures, study, and examination as to insist that undergraduates working for an Honours degree should first devote three years to working for a mere pass. The nature of the examination to which they are subjected in the end is not the main matter. If it were, it would be easy to add a few questions which better instructed candidates could answer if they desired. This is the method adopted at the local examinations, and it may do very well in subjecting candidates to tests in order to discover whether they are fitted to take a higher course.

But the point is that well-educated women ought to be put through a course differing in many particulars from that adapted to the abilities of women whose faculties have been less well cultivated. There must be no mistake about this. Nothing is more humiliating and worrying to a girl with a good education than to be treated to little snippets of easy physiology, and kept grinding away for months at subjects which under a better system she could master in a week or two. She learns little, becomes discouraged, and ends by being stale. The remedy, so far as we can see, is for educated women to be received in training schools able to maintain a certain standard of education for their candidates. The course could then be adapted to their understanding. The theoretical part of the simple pass examination could perhaps be taken at the end of the second year, though of course actual registration must be deferred until the three years' term of training were completed. The third and fourth years could be devoted to higher subjects, in affiliation with the local university, and would lead on naturally to the special university course which would crown the whole.

It will not do to be obsessed with a convention about uniformity. In all practical matters every nurse must, of course, go through the mill in the same way. But as regards theory distinctions must inevitably be observed. The practical nurse, who aims at no more than a subordinate post in a profession which offers fifty or a hundred such posts to one of a higher grade, happily needs only an elementary grasp of technicalities. With this she may aspire to some of the highest prizes in her profession from a monetary point of view, may be loved and honoured by all with whom she works, may set the name of nurse very high among her patients, and make part of one of the strongest forces for the national welfare. It is the glory of trained nursing that this is so.

But women with well-developed reasoning powers, retentive memory, and aptitude for languages, quick to understand and accurate to reproduce, will respond all the better when high demands are made upon them. The lectures, the text-books, and the methods found suitable for the one class of nurse are not successful with the other. To subject them to identical examinations, even with a few starred questions, is fair to neither.

